# TaxMapper: an analysis tool, reference database and workflow for metatranscriptome analysis of eukaryotic microorganisms

**DOI:** 10.1186/s12864-017-4168-6

**Published:** 2017-10-16

**Authors:** Daniela Beisser, Nadine Graupner, Lars Grossmann, Henning Timm, Jens Boenigk, Sven Rahmann

**Affiliations:** 1 Biodiversity, University of Duisburg-Essen, Universitätsstr. 5, Essen, 45141 Germany; 2 Genome Informatics, University of Duisburg-Essen, University Hospital Essen, Hufelandstr. 55, Essen, 45147 Germany

**Keywords:** Metatranscriptome analysis, Taxonomic assignment, Protists

## Abstract

**Background:**

High-throughput sequencing (HTS) technologies are increasingly applied to analyse complex microbial ecosystems by mRNA sequencing of whole communities, also known as metatranscriptome sequencing. This approach is at the moment largely limited to prokaryotic communities and communities of few eukaryotic species with sequenced genomes. For eukaryotes the analysis is hindered mainly by a low and fragmented coverage of the reference databases to infer the community composition, but also by lack of automated workflows for the task.

**Results:**

From the databases of the National Center for Biotechnology Information and Marine Microbial Eukaryote Transcriptome Sequencing Project, 142 references were selected in such a way that the taxa represent the main lineages within each of the seven supergroups of eukaryotes and possess predominantly complete transcriptomes or genomes. From these references, we created an annotated microeukaryotic reference database. We developed a tool called TaxMapper for a reliably mapping of sequencing reads against this database and filtering of unreliable assignments. For filtering, a classifier was trained and tested on each of the following: sequences of taxa in the database, sequences of taxa related to those in the database, and random sequences. Additionally, TaxMapper is part of a metatranscriptomic Snakemake workflow developed to perform quality assessment, functional and taxonomic annotation and (multivariate) statistical analysis including environmental data. The workflow is provided and described in detail to empower researchers to apply it for metatranscriptome analysis of any environmental sample.

**Conclusions:**

TaxMapper shows superior performance compared to standard approaches, resulting in a higher number of true positive taxonomic assignments. Both the TaxMapper tool and the workflow are available as open-source code at Bitbucket under the MIT license: https://bitbucket.org/dbeisser/taxmapperand as a Bioconda package: https://bioconda.github.io/recipes/taxmapper/README.html.

**Electronic supplementary material:**

The online version of this article (doi:10.1186/s12864-017-4168-6) contains supplementary material, which is available to authorized users.

## Background

### Motivation and goals

Metatranscriptome sequencing of diverse ecosystems is becoming a common methodology in many research institutions, and large scale sampling campaigns such as the Marine Microbial Eukaryote Transcriptome Sequencing Project (MMETSP, [1]) and the Tara Oceans expedition [2] have contributed to a growing amount of available environmental sequencing data. However, the analysis of the resulting short read sequences is still far from routine, especially for unicellular eukaryotic organisms, due to what was termed by Escobar-Zepeda et al. as “the neglected world of eukaryotes in metagenomics” [3]. This is particularly severe since microscopic eukaryotes (protists) constitute a paraphyletic taxon [4] spread over the whole eukaryotic tree of life and represent the bulk of most major groups, whereas multicellular lineages are confined to small corners [5]. Protists occur at high abundance in almost all habitats, e.g. in freshwaters, oceans, biofilms and soils [2, 5–9]. They maintain ecosystem functions, as they are responsible for most planktonic primary production [10], are the most important feeders of bacteria [7, 11] and key players in the regulation of element cycling, particularly carbon [7, 12].

Perhaps surprisingly then, protists are poorly covered by genomic reference databases despite their broad diversity, and if at all, only few model species are present. Therefore, most recent metatranscriptome approaches were designed for prokaryotes, which offer more complete databases (e.g. NCBI) in contrast to eukaryotes. Here, efficient mapping approaches, such as BWA or Bowtie, and methodologies allowing few differences to the reference sequences (e.g. k-mer indices) can be used. It is frequently possible to obtain taxonomic assignments even down to species level.

In contrast, few genome sequences from eukaryotes exist, and those that do are not well balanced across the main lineages of the eukaryotic tree of life, and therefore do not reflect the diversity within these lineages. The main focus of publicly available genomes lies on the Opisthokonta (Fungi/Metazoa group), including many animals, in particular model organisms, and Viridiplantae (green plants, containing Streptophyta and Chlorophyta) with an emphasis on crop plants. For example, in the NCBI database the available genomes in these two groups already represent 96% of the available genomes for eukaryotes, whereas eukaryotic genomes represent 43% of all genomes from the three domains (bacteria: 54%, archaea: 3%, NCBI June 2017).

The diversity of microbial eukaryotes is strongly underrepresented and database searches that aim at an assignment of metatranscriptomic reads on species level will, for the most part, be incorrect. This is caused by the fact that neither the species nor a close relative are included in the database and by the disproportional coverage of taxonomic groups leading to misassignments of reads to incorrect taxa by chance. In addition, available databases are often too large to be used in their entirety to map or search with millions of metatranscriptomic sequences on the read level.

A possible way out (taken here) is to restrict the taxonomic assignment to broader taxonomic groups, using appropriate reference organisms for each group. In turn, this requires a different approach to the similarity search, allowing to find more distantly related sequences. Since such similarity search tools are more time consuming, a reasonable search time can only be obtained by restricting the analysis to smaller reference databases.

Many existing approaches base their taxonomic assignments on selected sequenced marker genes. However, for a joint taxonomic and functional analysis (which taxonomic group performs which functions?), it is necessary to assign *each single read* to a taxonomic group and to a protein family.

Our goal was therefore to design, test and provide a comprehensive tool and workflow for eukaryotic metatranscriptome analysis, encompassing everything from preprocessing to integration of environmental data. A large impediment, as already mentioned, was a missing reference for the taxonomic assignment of sequences, which we constructed for all major taxonomic groups based on 142 publicly available transcriptomes and genomes. Our tool TaxMapper assigns taxonomic information to each read by mapping to the database using a reduced amino acid alphabet, and subsequently filtering of unreliable assignments. It is part of an automated rule-based Snakemake workflow developed to perform quality assessment and both functional and taxonomic annotation, as well as (multivariate) statistical analysis including environmental data.

In this work, we (i) describe the microeukaryotic reference database, (ii) present the TaxMapper software for taxonomic mapping and filtering of reads, and (iii) provide a detailed step-wise instruction on how to analyse metatranscriptomes from eukaryotic microorganisms using a modular workflow.

### Related work

Many metagenomic or metatranscriptomic analysis tools and workflows were conceived for the analysis of bacterial communities, like Leimena et al. [13], CLARK [14, 15], GOTTCHA [16], Genometa [17], MetaPhyler [18] or COMAN [19]. Others use a subset of the sequences for taxonomic profiling of metagenomes, such as MG-RAST [20], MetaTrans [21] and EBI metagenomics [22] that analyse rRNA and mRNA in samples. MetaPhlAn2 [23] and mOTU [24] use a subset of marker genes for taxonomic profiling and QIIME [25] uses Operational Taxonomic Units (OTUs) to assign a taxonomy. Recent k-mer based approaches such as Kraken [26], LMAT [27] or DUDes [28] need a user-specified library of genomes of species that are known to be present in the samples. The last category of tools searches the NCBI database to assign reads to taxonomical level after a BLAST-like search, including MEGAN [29], SAMSA [30] and Taxator-tk [31] or after a mapping with Bowtie2, e.g. Centrifuge [32].

## Implementation

### Reference database

To counter-balance the uneven diversity of eukaryotic microorganisms present in public databases, we construct the TaxMapper reference database such that it evenly includes genomic and transcriptomic sequences from all eukaryotic supergroups and taxonomic groups.

**Fig. 1:**
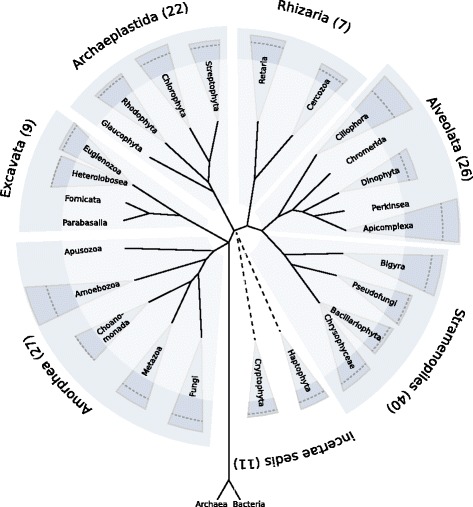
Taxonomy of eukaryotes. Taxonomy of eukaryotes with the supergroups and groups used in the reference database. Two remaining groups combining small lineages are not depicted. Coloured with darker background is the diversity of the supergroups and groups computed as the maximum Bray-Curtis dissimilarity over 4-mer spectra from the proteins of the reference genomes, as defined in [61]. Additionally, the mean Bray-Curtis dissimilarity is indicated as a dashed line. The taxonomy is based on Boenigk and Wodniok [53]

References from the databases of NCBI [33] and the Marine Microbial Eukaryote Transcriptome Sequencing Project [1] were selected based on the following criteria: (i) The taxa represent the main lineages within each of the seven supergroups of eukaryotes (see Fig. 1). (ii) Their genomes or transcriptomes are mostly complete; i.e., we excluded obviously incomplete datasets that consisted of only some hundred sequences. We thus selected 142 transcriptomes and genomes; the selection is described under “Results”.

The protein sequences of all reference genomes or transcriptomes were downloaded, redundant sequences were discarded for each species and the amino acid sequences were used to build a database index.

### TaxMapper

TaxMapper is designed to search sequence reads against remotely similar hits in the compiled database and to filter out hits of questionable certainty. It consists of five modules (search, map, filter, count, plot) that can be run individually with user defined parameters or as a single step with default settings.

The initial *search* in the indexed database is conducted for a single read file or forward and reverse reads in parallel using the protein similarity search tool RAPSearch2 [34] (v2.24, fast mode, using a loose *E*-value cutoff of 10^5^, but restricted to the best 20 hits). RAPSearch2 performs a fast similarity search in a reduced amino-acid search space. The best 20 hits are returned for each query (read) sequence and *mapped* to the 7 taxonomic supergroups and 28 main lineages. Two hits are kept subsequently, the best hit (BH) and the next best hit, according to *E*-value, that falls into another lineage (next lineage hit, NLH). (Hits that are better than the NLH and agree with the taxonomic group of the BH are skipped). Forward and reverse results can be combined by choosing either the option “best” to use the better of both searches or “concordant”, where forward and reverse have to map to the same taxonomic group.

**Fig. 2:**
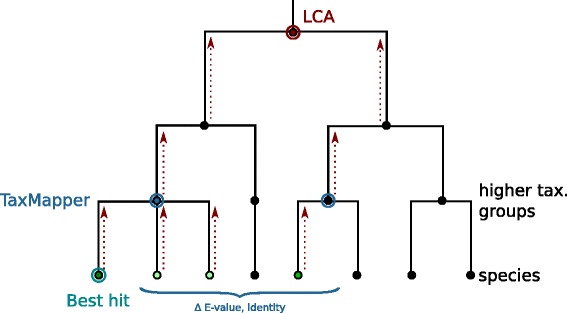
Differences between TaxMapper, LCA and best hit. Given the green leaves as possible hits, with the best hit circled in green, TaxMapper compares the best hits on a higher taxonomic level (blue circle) and uses the better hit (blue node) if the differences between the hits are large enough, while LCA is a bottom-up method that possibly returns the root of the taxonomy (red node) if the hits are too diverse

The *filter* idea behind TaxMapper is to assign taxonomic information only if the NLH is considerably worse than the BH. This means that only if the differences between BH and NLH in mapping properties such as the *E*-value, identity, alignment score etc. are large, the assignment of the BH is regarded trustworthy and is returned; otherwise no taxonomic group is ascribed to reduce wrong assignments. The details of the filter approach are discussed below (Subsection Filtering). Figure 2 illustrates the difference of this approach to other approaches that use only the best hit or the lowest common ancestor (LCA) of several hits. While the best hit approach returns just the best hit, regardless of further results that might be equally good, the lowest common ancestor approach returns the lowest level in the taxonomic tree that the hits have in common, which might be close to the root if the hits are too diverse.

Subsequently, *count* matrices can be generated over samples, summarizing the reads for all taxonomic groups to apply total count normalization and *plot* community compositions.

**Fig. 3:**
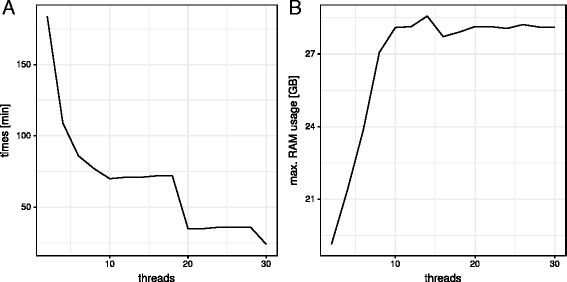
Performance curve on silver test dataset. (**a**) Processing time in minutes in dependence of used threads, from 2 to 30. (**b**) Maximum RAM usage in dependence of used threads

TaxMapper is implemented as a stand-alone tool in the Python language (v3.5). The statistical model for the filtering step (described below) was estimated using the generalized linear model function in R, applying maximum likelihood estimation (MLE). However, R is not required for running the TaxMapper software. TaxMapper can be run either stepwise with user-defined settings or for easier handling in one analysis step with default parameters. In the second case, just a folder of raw data in FASTQ or FASTA format has to be provided and all results are generated automatically. The analysis can be parallelized by declaring the number of threads to use and it is suggested to run it on a multicore machine, compute cluster or server for large datasets. In principle, it also runs on a recent desktop computer or laptop with a quad-core CPU and 16 GB RAM, but it is highly recommended to use more cores and resources for faster analysis. To provide an estimate on the processing time we report the times on the holdout dataset using our setting with 20 threads. For this dataset with 200 000 read pairs, all steps of TaxMapper take 32:49 minutes (wall clock time) on a server with AMD Opteron processors (6176, 2.3 GHz) and 500 GB of RAM. This corresponds to a user time (single thread) of 182:18 minutes, whereof the search step takes longest with 180:23 minutes. The 500 GB of available RAM were not fully used. As mentioned above, 16 GB of RAM are sufficient to run TaxMapper using 4 threads. It is recommended to have enough storage available, if intermediate results should be kept, since the mapping files contain up to 20 hits per reads in the worst case increasing the file size by a factor of up to 20. Running times and maximum RAM usage are additionally reported in dependence of the number of threads for the silver test dataset comprising 6 samples of 100 000 read pairs each, provided with TaxMapper, see Fig. 3. The analyses were performed on the same server as stated above. All threads were passed to RAPSearch running in multi-threaded mode, samples were analysed consecutively. In the provided workflow the user can decide how to split up the threads, whether to run several samples in parallel or provide the threads to RAPSearch. For samples run in parallel the database needs to be loaded repeatedly, which will increase the RAM usage.

### Filtering

The filtering step based on the best hit (BH) and the nearest lineage hit (NLH) is a distinguishing feature of TaxMapper. Since we found it impossible to separate correct from incorrect taxonomic assignments based on BH and NLH E-values alone, we estimated a logistic regression model based on five BH/NLH properties:

1. percent identity of the BH,
2. ratio of percent identity between BH and NLH,
3. log10*E*-value of BH,
4. difference in log10 E-values of BH and NLH,
5. the total size (in basepairs) of the BH’s taxonomic group in the database

The taxonomic group size was added as an independent variable in addition to the alignment statistics (*E*-value and identity) to include the different number of sequences per taxonomic group, which can bias hits toward more abundant taxa in the reference database.

In general, the binary logistic model is used to estimate the probability of a binary response *y*∈{0,1}, based on one or more independent variables (*x*_1_,…,*x*_*p*_):

1 $$ P(y = 1 \,|\, x_{1},\ldots,x_{p}) = 1/(1+e^{-(\beta_{0}+\sum_{k} \beta_{k} x_{k})})  $$

Here the *x*_*k*_ are the five hit properties described above, and *y*=1 corresponds to the event that the BH is a correct assignment, whereas *y*=0 means that the BH is an incorrect assignment. The goal is to search for values of the coefficients *β* such that the probability *P*(*y*=1 | *x*) is large when the hit properties *x* indicate that BH and NLH are sufficiently different such that the taxonomic assignment based on the BH is correct.

For estimating and testing the classifier, reads were chosen from 18 species that are included in the reference database and 17 species that are not included in the database, but where the taxonomic lineage is known and present in the database. Not all of the 28 groups could be used, since for some groups all available species were included in the database and further species for testing were not obtainable.

**Fig. 4:**
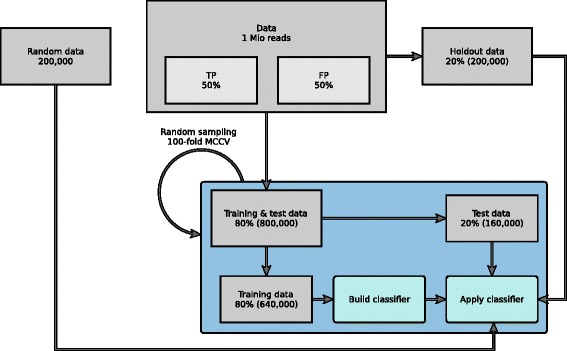
Classification scheme. One million reads from different taxonomic groups with 50% false positive and 50% true positive best hit assignments were used. This dataset was split in 20% holdout data and 80% training and test data, of which again 80% were used to train and 20% to test the classifier applying 100-fold Monte Carlo cross-validation. In addition, in each fold the holdout data and randomly simulated (nonsense) reads were used to evaluate the classifier

We obtained raw transcriptomic reads, listed with accession number in the Additional file 1. These were paired-end sequenced on an Illumina sequencer with a read length between 50 and 101 bp. Since for these reads, we know the correct taxonomic origin, we sorted them into two classes based on TaxMapper’s best hit (BH) alone: correctly classified or misclassified. We randomly chose 500 000 correctly classified (true positive, TP) and 500 000 misclassified (false positive, FP) reads as training data for estimating the model (see Fig. 4). This dataset of one million reads was split into 20% holdout data and 80% training and test data. The training and test data was again randomly split into 80% training and 20% test data 100 times to train and evaluate the classifier using 100-fold Monte Carlo cross-validation. In addition, in each cross validation round, the holdout data and randomly created reads were used to evaluate the classifier. Performance on the random reads (which by definition have no relation to any database sequence) allows us to estimate how well we are able to reject sequences that are from none of the eukaryotic lineages contained in the database. Results are given in the “Results” section.

### Workflow

A comprehensive workflow for metatranscriptome analysis was developed and made available in an executable Snakemake-based workflow. Snakemake is a workflow description language and execution environment developed by Köster et al. [35]. The workflow steps are defined in terms of rules with input, output and Shell, Python or R code. Dependencies between rules are automatically resolved and rules are automatically parallelized where possible. It features an easy to read, self-documenting syntax which also serves for version and parameter tracking. For the described workflow Snakemake version 3.9.1 was used.

The workflow covers both taxonomic assignment of each read (using TaxMapper) and functional assignment (using RAPSearch2 on the UniProt database). Steps and parameters can be adjusted using a provided configuration file (config.yaml). The execution of each analysis step can be “turned on/off” by stating the output of the rules as input in rule all. Currently all outputs are added to the rule all to run a complete analysis.

**Fig. 5:**
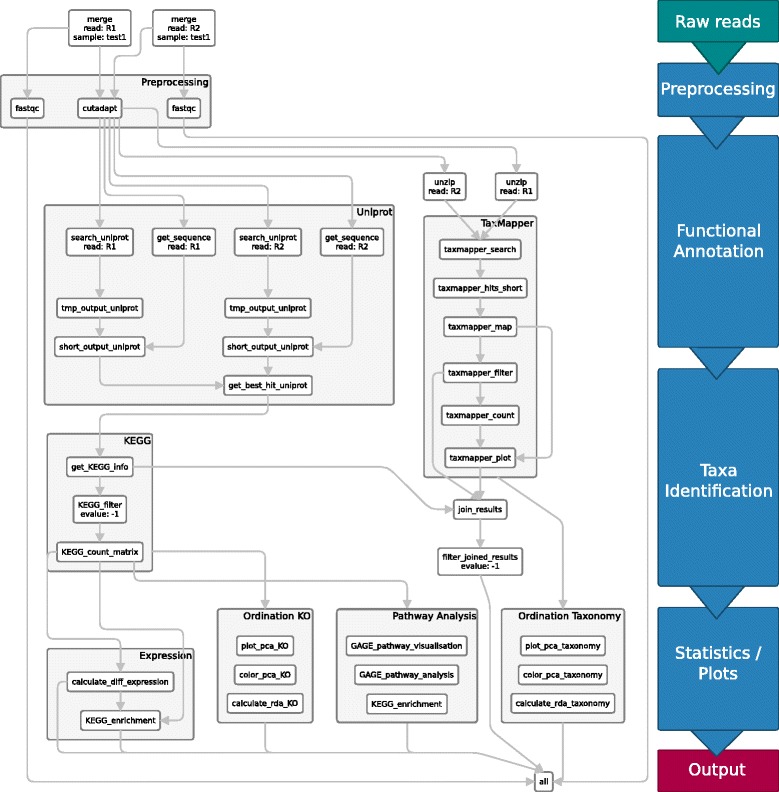
Snakemake workflow. Rules of the Snakemake workflow processing the FASTQ input files to the final output *all*

In the following, the most important rules and steps of the workflow are explained. An overview is given in Fig. 5.

The steps of the bioinformatic workflow are specified in the workflow management system Snakemake. Snakemake rules describe how to create output files from input files by executing commands on the input files. The commands can also be run on single files in the terminal, Python or R, but for automation, parallelization and reproducibility of the workflow, Snakemake is used. We briefly explain the Snakemake syntax here on a short example Snakemake file:



The desired final outputs of the workflow are described in the rule all, these are “plots/dataset1.pdf” and “plots/dataset2.pdf”. To create the plot, we run a shell command in the rule create_plots on the input “raw/{dataset}.csv” to create the output “plots/{dataset}.pdf”. Snakemake determines the rule dependencies by matching file names and automatically fills the wildcard dataset with the correct names: dataset1 and dataset2, that are expected as the input of rule all.

#### Preprocessing

The quality of raw sequencing reads is analysed using the quality control tool FastQC [36]. It computes various quality measures such as the base quality, overrepresented sequences, read length et cetera. The compressed FASTQ files are used as input and the snakemake rule runs FastQC as a shell command on the input. The wildcards sample and pair represent the sample name and forward and reverse read respectively.



Identified low quality bases and sequencing adapters can be removed with trimming tools such as cutadapt (v1.12, [37]). From the forward and reverse read, given as input, the adapter beginning with ‘GATCGGAAGAGCA’ and bases with a quality value below 20 are trimmed. If the remaining read length is below 50, the whole read will be discarded. All output files are saved in the folder results/cleaned.



#### Taxa identification

TaxMapper is used for the assignment and filtering of taxonomic information. For brevity, the one-step version is shown below, since it just needs an input folder with all FASTQ files and parallelization is performed within TaxMapper (here 20 threads are used via option -t). We have to get the input folder from the input files and provide an output file from TaxMapper as output for snakemake. The expand command is used to get a list of all input files by filling in the wildcards for sample and pair, which are lists of all filenames and forward and reverse reads provided in the configuration file. The database index is created within the subworkflow taxonomy which is given as the input database. To let Snakemake handle parallelization and provide user-defined parameters, the workflow can also be run in five successive steps: search, map, filter, count and plot (see Fig. 5 TaxMapper box).



#### Functional annotation

RAPSearch (v2.24, [34]), a fast protein similarity search tool, is used to search the read sequences in the Uniprot database (release 2016_06) [38]. The Uniprot database is downloaded and indexed as part of the workflow (in a subworkflow termed uniprot). The similarity search is performed with default parameters and the best hit is returned. Via a Uniprot identifier mapping file, obtained from the Uniprot database, KEGG (Kyoto Encyclopedia of Genes and Genomes, [39]) Orthology identifiers can be assigned to the query sequence.

Additional rules are used to shorten the output and combine the forward and reverse read mapping (see Fig. 5 Uniprot box). The input FASTQ files have to be first extracted from the gz archive to use them as input for RAPSearch2, then they are searched against Uniprot returning the alignments of the best hit or no result for each read.



#### Statistics and downstream analysis

Subsequent statistical analyses depend on the type of study and question. Since it is not always possible or intended to perform e.g. differential expression analysis, we included several possible rules in the workflow. All of the rules execute R code that is longer than a couple of lines and therefore not depicted here.

Existing rules include a differential expression analysis given different conditions using the Bioconductor package edgeR (v3.14.0, [40]), ordination analyses such as principal component analysis and redundancy analysis using the R package vegan (v2.3-4, [41]) and KEGG pathway analyses with the R packages GAGE (v2.21.1, [42]) and pathview (v1.9.0, [43]).

## Results

### Reference database

According to our criteria, 142 reference sequences were selected for the TaxMapper reference database (for details see Additional file 2). These references belong to the seven supergroups of eukaryotes, including 28 main lineages. In accordance with the taxonomy published by Boenigk and Wodniok [44] and with the tree of life project [45], we chose different levels of each lineage to cover their molecular and functional diversity. Figure 1 and Table 1 give an overview.

**Table 1:** Number of taxa in used taxonomic groups

Supergroup	Group	Number of taxa
Alveolata		**26**
	Apicomplexa	4
	Chromerida	2
	Ciliophora	8
	Dinophyceae	11
	Perkinsea	1
Amorphea		**27**
	Amoebozoa	7
	Apusozoa	1
	Choanoflagellida	2
	Fungi	6
	Metazoa	9
	Opisthokonta Rest	2
Archaeplastida		**22**
	Chlorophyta	12
	Glaucocystophyceae	2
	Rhodophyta	3
	Streptophyta	5
Excavata		**9**
	Euglenozoa	4
	Fornicata	2
	Heterolobosea	2
	Parabasalia	1
Hacrobia		**11**
	Cryptophyta	4
	Haptophyta	7
Rhizaria		**7**
	Cercozoa	3
	Foraminifera	4
Stramenopile		**40**
	Bacillariophyta	15
	Bigyra	4
	Chrysophyceae	6
	Pseudofungi	3
	Stramenopile Rest	12

Bold numbers: number of taxa used for each supergroup; non-bold: number of taxa used for each taxonomic group in the reference database

The supergroup Amorphea consists of two main lineages, the Opisthokonta (Holomycota and Holozoa) and Amoebozoa. Additionally, the small phylum Apusozoa is considered as a likely paraphyletic sistergroup of the Opistokonta [46, 47]. In the database the Amorphea are represented by 27 reference taxa. 19 taxa are affiliated with the Opisthokonta, including fungi representing the Holomycota, and Eumetazoa, Choanoflagellida (Choanomonada) and basal Opisthokonta, e.g. Filastera and Ichthyosporea here called Opisthokonta Rest, as representatives for the Holozoa. The Amoebozoa contain 7 reference taxa including lobose Amoebae, Archamoebae and Mycetozoa (slime moulds). One reference taxa is included for the phylum Apusozoa.

The supergroup Excavata is a very diverse group that can be summarized into two main groups, the Discoba including the lineages Euglenozoa, Heterolobosea and Jakobida as well as the Metamonada including the lineages Parabasalia and Fornicata. Many species of this supergroup are parasites [5] but some taxa e.g. most Euglenida are free-living and often occur in freshwater [48]. In the database the Excavata are represented by 9 reference taxa affiliated with Euglenozoa, Heterolobosea, Parabasalia and Fornicata. Due to few available transcriptomes of this supergroup in public databases and the focus on free-living taxa, only few references could be added.

The supergroup Archaeplastida includes three main lineages, the species-poor Glaucophyta (Glaucocystophyceae), the mostly marine Rhodophyta and the species-rich Viridiplantae (Chlorophyta, Streptophyta). Particularly the Chlorophyta are important primary producers in freshwater habitats [49]. Therefore, Archaeplastida are represented by 22 reference taxa affiliated with Chlorophyta, Streptophyta, Rhodophyta and Glaucocystophyceae.

The supergroup Rhizaria is a diverse group and consists of two main lineages, Cercozoa and Retaria (Foraminifera and Radiolaria). Cercozoa are very abundant in soil but can also occur in freshwaters and marine habitats [50]. In the database Rhizaria are represented by only 7 taxa belonging to Cercozoa and Foraminifera as there are only a few sequenced species available in public databases, particularly from Cercozoa.

The supergroup Alveolata is a very diverse group. It consists of three main lineages, Ciliophora, Apicomplexa and Dinophyta. Further, the smaller lineages Chromerida, Colpodellids and Perkinsea are affiliated with the Alveolata. Ciliophora and Dinophyceae can occur in high abundances and are important predators of other protists [51, 52]. Due to their importance and diversity they are covered by a high number of reference taxa (26) in the database: Ciliophora, Apicomplexa, Dinophyceae, Chromerida and Perkinsea.

The supergroup Stramenopiles is a very diverse group including many lineages which can be summarized into three groups, the Pseudofungi, the heterotrophic Bigyra and the plastid bearing Ochrophyta [53]. Some of these lineages, e.g. Bacillariophyta and Chrysophyceae, are very abundant in freshwater habitats [49, 51]. They are important primary producers and predators of bacteria. Therefore, we covered this group by a high number of 40 reference taxa. Pseudofungi were included as well as Bigyra summarizing the three lineages Bicosoecida, Blastocystis and Labyrinthulida. The Ochrophyta are represented by the two abundant freshwater groups Bacillariophyta and Chrysophyceae and a collection of other reference taxa affiliated with several Stramenopile lineages called Stramenopiles Rest.

An additional “group” in the eukaryotic tree of life are the incertae sedis Eukaryota which include amongst others the Hacrobia (Cryptophyta, Haptophyta) [5]. The evolutionary position of theses taxa is still uncertain as the phylogenetic position differs depending on the studied organism and genes. In the database Hacrobia are represented by 11 reference taxa, affiliated with Cryptophyta and Haptophyta.

The database is available as a FASTA file in a separate Bitbucket repository at https://bitbucket.org/dbeisser/taxmapper_supplement/src/master/databases/taxonomy/.

### Evaluation of the filtering step

After training the classifier to reject assignments of training reads whose best hit misses the correct taxonomic group, we evaluated the performance on the test, random and holdout dataset.

**Fig. 6:**
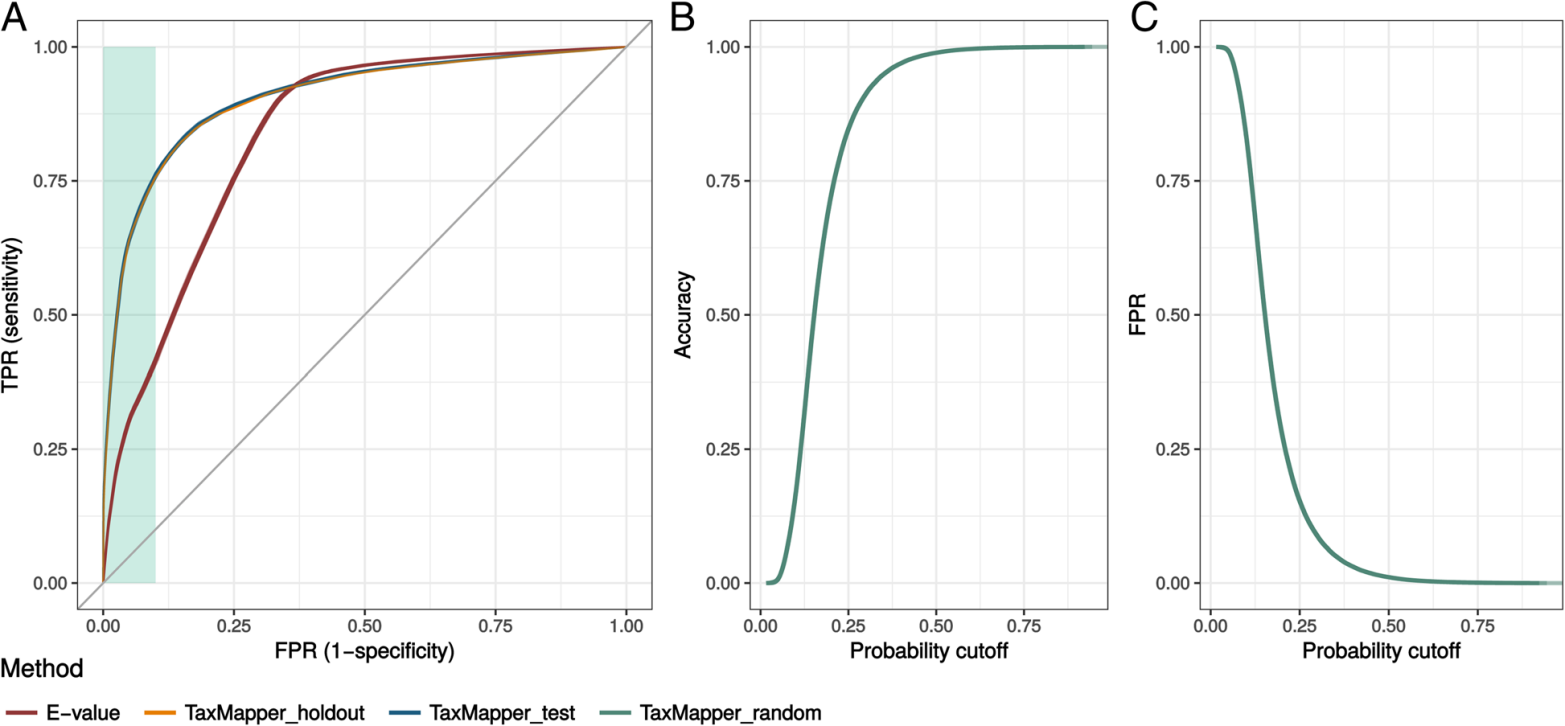
Validation. (**a**) False positive rate (FPR, x-axis) versus true positive rate (TPR, y-axis) of TaxMapper results on test dataset (blue), TaxMapper result on holdout data (orange), and simple *E*-value cutoff results on test dataset (red). (The blue and orange curves overlap in the subfigure on the left side.) The green background indicates the desired area with a low FPR (≤0.1). (**b**) Accuracy of TaxMapper on the random nonsense data (green) against the probability cutoff. (**c**) False positive rate of TaxMapper on the random data over all probability cutoffs

The results are depicted as receiver operating characteristic (ROC) curves in Fig. 6 A and compared based on the area under the curve (AUC) and accuracy (ACC) in Table 2. Shown are true positive rate (TPR) and false positive rate (FPR) of TaxMapper results varying over the cutoff for the probability *P*(*y*=1|*x*_1_,…,*x*_5_). Results are also given when no logistic model, but a simple *E*-value cutoff for the best hit, is used.

**Table 2:** Evaluation of TaxMapper. Comparison of area under the ROC curve (AUC) and accuracy (ACC) for the *E*-value cutoff (test data) and TaxMapper on test, holdout and random data. The cutoffs leading to the best results in ACC and a false positive rate below 0.1 are shown below

Method	Simple *E*-value cutoff	TaxMapper test	TaxMapper holdout	TaxMapper random
AUC	0.84	0.91	0.90	NA
ACC	0.79	0.84	0.84	1.00
Cutoff for best ACC	-0.92	0.38	0.40	1.00
Cutoff for FPR < 0.1	1.66	0.58	0.58	0.29

TaxMapper yields superior results, especially in the desired area with low false positive rates, and an AUC of 0.90–0.91 in contrast to 0.84 for the simple *E*-value cutoff method. The highest accuracy of 0.84 was obtained for a probability cutoff of 0.38 and 0.40 for TaxMapper (test and holdout data, respectively). The best accuracy (0.79) for a simple *E*-value cutoff lay below −0.92 (log10*E*-value).

A false positive rate below 0.1 could be obtained with a probability cutoff of 0.58 or log10*E*-value below 1.66. Obviously, in the random dataset only the number of false positives can be reduced, resulting in the best accuracy of 1.0 for a probability cutoff of 1.0, filtering out all reads. But as shown in Fig. 6 b and c, the accuracy increases rapidly and a low false positive rate below 0.1 is already obtained with an average probability cutoff of 0.29 (see Fig. 6 and Table 2).

### Evaluation of TaxMapper against other tools

**Fig. 7:**
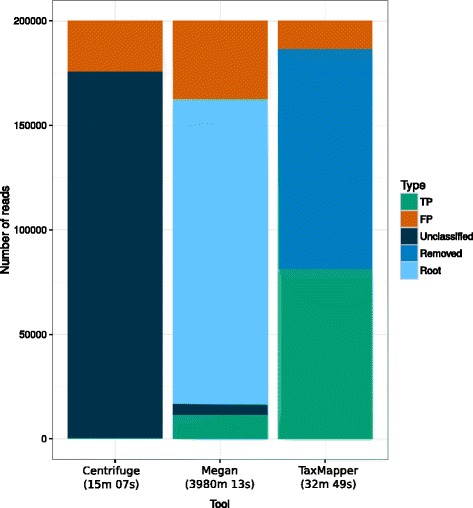
Comparison to other tools. Shown are the results obtained on the holdout dataset using the tool Centrifuge, TaxMapper and Taxator-tk with the Megan algorithm and the required wall clock time in brackets (run with 20 threads in parallel). Depicted are the number of reads resulting in a true positive (TP) assignment, false positive (FP) assignment, unclassified taxonomy, reads mapping to the root of the taxonomic tree and filtered (removed) reads by TaxMapper

The processing time and results of TaxMapper were compared to the tools Taxator-tk [31] and Centrifuge [32], to our knowledge the only tools that can be run on a server and assign sequences to a taxonomy on read-level (see Fig. 7). Both tools were run with default parameters and as described in the manual. The non-redundant NCBI index was used as a reference for Centrifuge as provided by the authors. For Taxator-tk the provided refpacks could not be used, since they focus on prokaryotic taxa, therefore a refpack using the NCBI nr database was built according to the instructions on the website. The search step of Taxator-tk utilises a blastn or LAST [54] search against the NCBI non-redundant nucleotide database. Due to the long running time, only the holdout data with 200 000 reads was tested. Overall, Taxator-tk using the Megan algorithm [29] takes 3980:13 minutes, Centrifuge takes 15:07 minutes and TaxMapper 32:49 minutes (wall clock time) on a server with AMD Opteron processors (6176, 2.3 GHz) using 20 threads. This corresponds to a user time of 182:18 minutes for TaxMapper, of which the search step takes longest with 180:23 minutes. Centrifuge uses the fast mapping algorithm Bowtie2 [55] to map the reads against the NCBI database. The drawback is that Bowtie2 allows few mismatches and therefore reads map only to very similar sequences. If the organism or a close relative is not contained in the database, a taxonomy cannot be assigned, leading to many unclassified reads for this method. The Megan algorithm of Taxator-tk uses BLAST, therefore only few reads are unclassified, but the majority map to the root node of the taxonomy, due to the lowest common ancestor approach described in Fig. 2. The original algorithm developed for Taxator-tk is optimized for longer reads, starting with 500 bp, and was not used here. TaxMapper results in the highest number of true positive assignments and the lowest number of false positives. Results where the taxonomic assignment of the best hit was unresolvable, due to a low certainty and high similarity to another taxonomic group, were removed in the filter step.

### Example application: silver dataset

**Fig. 8:**
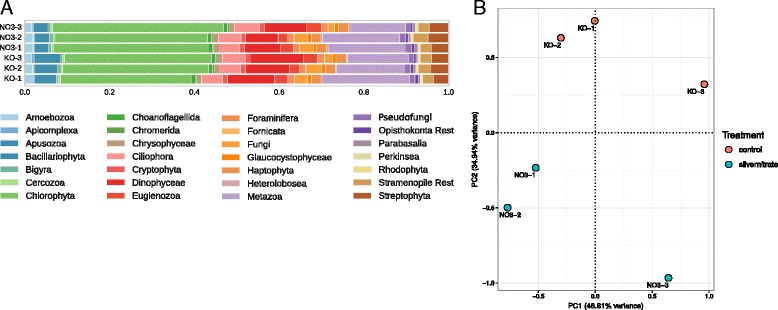
Community composition and principal component analysis of silver dataset. (**a**) Community composition of a subset of data from a metatranscriptome sequencing study, where the effect of silver nitrate was tested on the community and function. (**b**) Principal component analysis (PCA) of the TMM-normalized taxonomic count data, colored according to treatment: control in red and samples with added silver nitrate in blue

To showcase an application, the metatransciptome workflow was run on a subset of sequencing data from a study published in 2014 by Boenigk et al. [53]. In brief, a short-term silver exposure experiment was conducted on nine 20 L plastic tanks containing water from a natural plankton community from an eutrophic pond at the campus Essen of the University Duisburg-Essen. The nine tanks were divided into three experimental groups (control, silver nitrate and silver nanoparticle exposure) with three replicate tanks each. The subsample used here contains the control samples and the silver nitrate samples. The metatranscriptomic workflow was applied to analyse the functional and taxonomic differences between the treatments. Figure 8 A depicts the community compositions with the largest changes visible in the groups Bacillariophyta and Chlorophyta. The taxonomic changes are also depicted in the PCA in Fig. 8 B, separating on the second principal component the control samples from the samples treated with a sublethal silver concentration of 5 *μ*g/L. On the functional level a test for differential expression reveals 34 KEGG orthologous genes that differ significantly (FDR <0.1) between the two groups and show an enrichment of photosynthesis pathways. It is known that silver ions affect the primary metabolism in particular photosynthesis by direct interference [53, 56]. On the other hand, it has been shown that for low concentrations of silver green algae grows is increased as observed in Fig. 8 A [57].

A subset of this study with the first 100 000 reads per FASTQ file is provided with the workflow as test dataset.

## Discussion

### Related work

#### Metatranscriptome workflows

Existing metatranscriptome workflows often focus on bacterial composition: Leimena et al. [13] describe in detail an analysis pipeline for prokaryotic datasets. SAMSA [30] is also a metatranscriptome analysis pipeline which is mostly suited for prokaryotes because of its use of the NCBI RefSeq database. COMAN [19] maps metatranscriptome reads to bacterial reference genomes, and MetaTrans [21] assigns a taxonomy based on prokaryotic 16S rRNA.

Other studies construct pipelines for subparts of the analysis, including Goncalves et al. [58] who constructed an R-based pipeline for pre-processing, quality assessment and expression estimation of RNA sequence datasets, and Marchetti et al. [59] who provide an R package for differential expression analysis of metatranscriptome sequences starting from a count matrix of genes and a phylogenetic annotation. For our purposes, these approaches have two disadvantages: (i) they provide no complete executable workflow, or (ii) the available workflow parts cannot be easily adapted to eukaryotic data.

#### Metatranscriptome analysis tools

Similarly, many metagenomic or metatranscriptomic analysis tools were conceived for the analysis of bacterial communities. For example, CLARK [14, 15] is a tool for the taxonomic classification of metagenomic reads using known bacterial genomes. GOTTCHA [16] is a taxonomic profiler that uses non-redundant signature databases for prokaryotic and viral genomes. Genometa [17] is a Java program to identify bacterial species and gene content from high-throughput datasets. MetaPhyler [18] estimates bacterial composition from metagenomic samples.

Others use a subset of the sequences for taxonomic profiling of metagenomes. Web-based solutions are provided by MG-RAST [20] and EBI metagenomics [22] that automatically analyse rRNA and mRNA in submitted samples. MetaPhlAn2 [23] and mOTU [24] use a subset of marker genes for taxonomic profiling. QIIME [25] uses Operational Taxonomic Units (OTUs) to assign a taxonomy.

A user-specified library of genomes of species that are present in the samples has to be provided for recent programs utilizing k-mers such as Kraken [26], LMAT [27] or DUDes [28]. For environmental data, this is not possible, the programs are better suited for laboratory experiments with low-complexity communities of known species or strains or for the detection of specific organisms in a sample, e.g. related to a disease.

Another category of tools searches the NCBI database to assign reads to a taxonomic level after a BLAST search, including MEGAN [29] and Taxator-tk [31] or after a mapping with Bowtie2, e.g. Centrifuge [32].

For our purposes, we found that each existing tool exhibited a shortcoming that rendered it unsuitable for the read-level assignment of taxonomic and functional information to microeukaryotic sequences. We summarize our requirements versus the properties of existing tools in Table 3.

**Table 3:** Issues with properties of existing approaches. Properties of existing approaches versus requirements for microeukaryotic environmental sequence analysis

Property	Existing approaches	Requirements
Organisms	Prokaryotic	Eukaryotic
Taxonomic assignment from	Marker genes	All reads
Taxonomic assignment on	Species level	Higher taxonomic level
Type of tool	GUI, webservice	Stand-alone, workflow
Similarity to reference	High	Low
Search method	Mapping with BWA, Bowtie2	Variant-tolerant local alignment
Database size	Large	Small – midsize

The tools Taxator-tk and Centrifuge were selected for a comparison since they seemed to be the most suitable for our purposes. They directly work with mRNA reads and search the complete NCBI database, which in principle includes eukaryotic sequences. Additionally, two search strategies are represented by using them, BLAST and Bowtie2. We found that on the holdout dataset, their performance was low. Searches with Bowtie2 allow few mismatches, and therefore reads map only to sequences of closely related species which are rare for microeukaryotes in NCBI. The drawback of the Megan algorithm of Taxator-tk is a high assignment to the root node of the taxonomy, due to the lowest common ancestor approach and unspecific hits to sequences contained in NCBI. The original algorithm developed for Taxator-tk is optimized for longer reads, starting with 500 bp, and could not be used for Illumina reads.

### Limitations and recommendations

#### Other datatypes

The intended use-case for our tool and workflow are metatranscriptomic high thoughput sequencing studies for microeukaryotes. This implies that eukaryotic mRNA was obtained by RNA extraction and polyA selection. As a result, rRNA, prokaryotic mRNA and other small RNA should be removed or strongly reduced in the library. For low-complexity communities with known species, rRNA degradation could also be an option to remove rRNA from the samples and keep prokaryotic sequences, but from our experience this may lead to a high abundance of prokaryotic sequences with few eukaryotic reads. To use TaxMapper on such samples, we recommend to first filter out prokaryotic reads and then use TaxMapper on the remaining reads. Without splitting the dataset, the analysis will be more time-demanding and may lead to false assignments of prokaryotic reads to eukaryotic reference sequences. We assessed the performance with default parameters on prokaryotic metatranscriptome samples by using 200 000 reads from a simulated community containing bacteria, fungi and viruses by Jeremy Cox et al. [60]. 86% of the transcripts were removed and 2% assigned to micro-fungi; therefore without a strict cut-off we would have around 12% false assignments of prokaryotic sequences to eukaryotic references in this dataset.

Likewise, our tool was not intended for metagenome analyses. It is expected to perform well on protein coding regions, but due to the protein reference sequences, the taxonomic and functional assignment will fail for non-coding and intronic regions. Since it is out of the scope of this paper, we did not test the performance using metagenomic samples.

We also did not test the metatranscriptome analysis using long read data. Currently, we consider the sequence output of nanopore technologies as too low for metatranscriptome studies. In the future, this will likely change, and in principle, TaxMapper has no restrictions on the length of the reads. It is already possible to search with longer sequences and provide FASTA files as input.

#### Functional assignment

In contrast to the evaluation of the taxonomic assignment method, we did not rigorously test the other steps of the workflow, e.g. the functional assigment and statistical methods. Since the workflow combines well-known and commonly used methods, we refer to the original publications of the methods for an evaluation (see subsection Workflow in the Methodology). Concerning running time, the functional assignment takes about 20% of the time of the taxonomic assignment. If parts of the analysis workflow are not required, they may be “turned off” (see subsection Workflow in the Methodology) to save time. The functional analysis is currently limited to a search in the Uniprot database. Uniprot IDs, gene symbols and KEGG Orthology IDs are reported if these are available. The direct assignment of reads to KEGG pathways is only possible if a KEGG license is available. The create_kegg_mapping subworkflow creates a KEGG mapping if the path to a local KEGG database is provided, otherwise this part will be skipped. Please note, that the KEGG pathways analysis is based on the R packages gage and pathview, which retrieve necessary information from the KEGG database. A warning is issued upon execution that non-academic users may require a KEGG license agreement. Overall, with standard RAPSearch parameters we obtain for the silver dataset on average an assignment of 27.9% of the reads to Uniprot IDs, 23.7% to gene symbols and 16.6% to KO IDs. A higher coverage would also be desirable here, this will hopefully increase in the future with a rising number of sequenced and annotated eukaryotic genomes.

#### Database

When new sequences become available which further complete the diversity of the eukaryotic supergroups, an update of the database will be released. In particular, the Excavata and Rhizaria should be extended in future versions, for which at the moment only few appropriate genomes or transcriptomes are present. This might lead to a higher number of unassigned reads to these taxonomic groups. Missing lineages include representative taxa of the Jakobida (Excavata; Discoba), Radiolaria (Rhizaria; Retaria) and Colpodellids (Alveolata). Additionally, some lineages of the Eukaryotes incertae sedis e.g. Katablepharids and some small groups that have only few genera e.g. Nucleariids (Amorphea) are also not yet contained in the reference database.

## Conclusions

Despite the large number of tools developed for taxonomic analyses, the majority of them aims at different sequencing data (e.g. rRNA, contigs) or organismic groups (prokaryotes) and does not allow a combined functional and taxonomic analysis of metatranscriptomic data. We therefore developed the presented tool TaxMapper to work in conjunction with a constructed microeukaryotic reference database for taxonomic assignment, and included the taxonomic analysis in a complete workflow for metatranscriptomic sequence analysis.

The smaller, but more appropriate reference for protists, allows a faster search than a comparable search against whole NCBI.

False positive assignments can be filtered using a probability cutoff on a logistic regression model based on features of the best hit and next lineage hit, which yielded better results than a simple *E*-value cutoff.

TaxMapper can be run straightforwardly on a folder of sequencing data or as part of the Snakemake workflow. The workflow performs quality assessment, functional and taxonomic annotation and (multivariate) statistical analyses using available environmental factors or different sample groups. The provided workflow ensures a reproducible analysis which can be easily extended to new samples.

## Availability and requirements

The data and software are available at Bitbucket https://bitbucket.org/dbeisser/taxmapper, https://bitbucket.org/dbeisser/taxmapper_supplementand as a Bioconda package: https://bioconda.github.io/recipes/taxmapper/README.html.

- **Project name:** TaxMapper
- **Project home page:** https://bitbucket.org/dbeisser/taxmapper
- **Operating system(s):** Linux
- **Programming language:** Python
- **License:** MIT

## Additional files

Additional file 1 Validation taxa. Information on taxa used for evaluating the logistic regression model. (CSV 3 kb)

Additional file 2 Taxa contained in reference database. Information on taxa contained in reference database, including taxonomic affiliation, accession number and database. (CSV 14 kb)

## Abbreviations

ACC: Accuracy
AUC: Area under the curve
BH: Best hit
BLAST: Basic Local Alignment Search Tool
DFG: Deutsche Forschungsgemeinschaft
FDR: False discovery rate
FP: False postive
FPR: False positive rate
HTS: High-throughput sequencing
KEGG: Kyoto Encyclopedia of Genes and Genomes
LAST: Local Alignment Search Tool
LCA: Lowest common ancestor
MLE: Maximum likelihood estimation
MMETSP: Marine Microbial Eukaryote Transcriptome Sequencing Project
NCBI: National Center for Biotechnology Information
NLH: Next lineage hit
OTU: Operational Taxonomic Unit
PCA: Principal component analysis
ROC: Receiver operating characteristic
TMM: Trimmed mean of M-values
TP: True positive
TPR: True positive rate
